# Predictors of job satisfaction among nurses working in Ethiopian public hospitals, 2014: institution-based cross-sectional study

**DOI:** 10.1186/s12960-017-0204-5

**Published:** 2017-04-24

**Authors:** Ayele Semachew, Tefera Belachew, Temamen Tesfaye, Yohannes Mehretie Adinew

**Affiliations:** 1 School of Nursing, College of Medicine and Health Sciences, Bahir Dar University, Bahir Dar, Ethiopia; 2 Department of Population and Family Health, College of Public Health and Medical Sciences, Jimma University, Jimma, Ethiopia; 3 Department of Nursing, College of Public Health and Medical Sciences, Jimma University, Jimma, Ethiopia; 4 College of Health Sciences and Medicine, Wolaita Sodo University, Sodo, Ethiopia

**Keywords:** Job satisfaction, Nursing, Public hospital, Ethiopia, McCloskey/Mueller Satisfaction Scale

## Abstract

**Background:**

Nurses play a pivotal role in determining the efficiency, effectiveness, and sustainability of health care systems. Nurses’ job satisfaction plays an important role in the delivery of quality health care. There is paucity of studies addressing job satisfaction among nurses in the public hospital setting in Ethiopia. Thus, this study aimed to assess job satisfaction and factors influencing it among nurses in Jimma zone public hospitals, southwestern Ethiopia.

**Methods:**

An institution-based census was conducted among 316 nurses working in Jimma zone public hospitals from March to April, 2014. A structured self-administered questionnaire based on a modified version of the McCloskey/Mueller Satisfaction Scale was used. Data were entered using Epi Info version 3.5.3 statistical software and analyzed using SPSS version 20 statistical package. Mean satisfaction scores were compared by independent variables using an independent sample *t* test and ANOVA. Bivariate and multivariable linear regressions were done.

**Results:**

A total of 316 nurses were included, yielding a response rate of 92.67%. The overall mean job satisfaction was (67.43 ± 13.85). One third (33.5%) of the study participants had a low level of job satisfaction. Mutual understandings at work and professional commitment showed significant and positive relationship with overall job satisfaction, while working at an inpatient unit and work load were negatively associated.

**Conclusions:**

One third of nurses had a low level of job satisfaction. Professional commitment, workload, working unit, and mutual understanding at work predicted the outcome variable.

## Background

Job satisfaction refers to the attitude and feelings people have about their work [1]. Stamps (1997) defined it as “it is the extent to which employees like their jobs [2]”. Job satisfaction is one of the most important factors that determine efficiency and productivity of human resources [3].

Health care is a labor-intensive industry [4], and the single-largest health care professional groups within it are nurses [5]. In recent years, a major target of the health care delivery system has been the provision of quality care. A fundamental challenge, however, still remains to achieve improved patient outcome [6].

As key members of the health care team, nurses’ job satisfaction plays an important role in the delivery of high-quality health care [7]. As the largest health care profession in the world [8], there is no doubt that nurses are key to the achievement of the Millennium Development Goals (MDGs) [9]. They are often the only health professionals accessible to many people in their lifetime, and addressing their satisfaction is a very important issue [10]. In Ethiopia, nurses are the backbone of the health care system [11]. They play a vital role in promoting the health care program of the country by contributing their own share for the achievement of the MDGs [12].

Nurses’ job satisfaction is a multidimensional phenomenon influenced by many variables [13]. Job dissatisfaction has frequently been cited as the primary reason for a high rate of absenteeism and turnover of nurses which pose a threat to a health care organization’s capacity to provide quality care by impeding their efficiency and effectiveness [14]. Nurses’ perceptions of the satisfaction with their jobs have positive impacts on customer-perceived service quality in health care services [15].

Nursing staffs in most medical institutions are the most undervalued staff members [16] despite the fact that these are the people who take care of our loved ones around the clock when they are hospitalized [17]. Currently, nurses seem to be less satisfied due to many unidentified factors, and hence, there is a constant threat of attrition [1]. The nursing profession is also unable to attract adequate number of men and women due to salaries, shift schedule, and social perception of nurses [18]; nowadays in Ethiopia, a number of nurses are leaving their profession and are trying to join other non-nursing fields [11]. Therefore, this study set out to assess the level of nurses’ job satisfaction and factors associated with it in Jimma zone public hospitals.

## Methods

### Study design and setup

An institution-based census was conducted from March to April, 2014, among 360 nurses with work experience of greater or equal to 6 months working in three public hospitals found in Jimma zone, namely Shenen Gibe, Limu Genet, and Jimma University Specialized Hospital. Jimma is found at 352 km southwest of Addis Ababa. Based on the 2007 Census, this zone has a total population of 2 486 155.

### Data collection tool and procedure

To collect the data, different tools were used in this study.

*Part I*: Socio-demographic questionnaires developed by the investigators

*Part II*: McCloskey/Mueller Satisfaction Scale (MMSS) having eight subscales:

(i) Extrinsic reward subscale consisting of three items
(ii) Scheduling subscale consisting of five items
(iii) Family and work balance subscale consisting of two items
(iv) Coworker subscale consisting of two items
(v) Interaction opportunity subscale consisting of four items
(vi) Professional opportunities consisting of four items
(vii) Praise and recognition consisting of four items and
(viii) Control and responsibility consisting of five items and these eight subscales have 29 items

The McCloskey/Mueller Satisfaction Scale (MMSS) was used to assess nurses’ job satisfaction [19]. Response options in this study ranged from 1 (*very dissatisfied*) to 4 (*very satisfied*) eliminating the neutral response option to encourage participants to make a decision regarding their thoughts and feelings as opposed to indicating that they had no opinion [20]. Questions in this study were developed from a previous study [21] with Cronbach’s alpha of 0.95. In the present study also, the Cronbach’s alpha coefficient was 0.90.

*Part III*: Nurse-physician communication-related factors having three subscales and items under each subscales were measured with a 5-point Likert scale. In the present study, it showed a Cronbach’s alpha value of 0.81.

(i) Personal factor (measured by three items)
(ii) Organizational factor (measured by six items)
(iii) Mutual understanding at work (measured by three items)

*Part IV*: Job stress-related factors having four subscales and items under each subscales were measured with a 4-point Likert scale. In the present study, it had a Cronbach’s alpha value of 0.81.

(i) Workload (measured by four items)
(ii) Lack of support (measured by three items)
(iii) Conflict (measured by four items)
(iv) Organizational decision (measured by three items)

*Part V*: Professional commitment factors having four items measured with a 4-point Likert scale. It has a Cronbach’s alpha value of 0.71.

And lastly, some open-ended questions were used to assess respondents’ reasons for being satisfied and dissatisfied.

Five diploma nurses were recruited to distribute, facilitate, and collect the data with close supervision.

### Data processing and analysis

The data were checked for completeness and entered into Epi Info version 3.5.3 statistical software and analyzed using SPSS version 20 statistical package. Descriptive statistics was used to describe the study population in relation to relevant variables.

Each item of the overall job satisfaction were measured by a 4-point Likert scale having a total of 29 items and their sum score ranging from a minimum of 29 to maximum of 116. For cut of value purpose, a data-driven classification system was used to classify the job satisfaction level into low, moderate, and high [22].

Mean satisfaction scores were compared by independent variables using an independent sample *t* test and ANOVA. After checking the assumptions, bivariate and multivariable linear regressions were done to see the association between the independent and the outcome variables. *β* coefficient was used to show independent predictors of satisfaction. Variables with a *p* value less than 0.05 were considered as statistically significant.

### Data quality assurance

Data quality was controlled by giving trainings and appropriate supervisions for data collectors. The overall supervision was carried out by the principal investigator. The questionnaire was pre-tested using 18 (5%) nurses that work in Woliso hospital. After analyzing the pretest result, appropriate modifications were made on the MMSS before the actual data collection.

## Results

### Socio-demographic characteristics of study participants

Out of the expected 341 respondents, 316 agreed to participate in the study, yielding a response rate of 92.7%. Majority of the participants (85.8%) work in Jimma University Specialized Hospital (JUSH). The mean age of participants’ was 27.87 (SD = 6.81) years. Almost half (49.4%) of the respondents were male. Nearly two thirds (62.3%) of the respondents were diploma holders. Regarding working units, 20.9 and 20.3% of them were from the surgical ward and outpatient department (OPD) respectively (Table 1).

**Table 1:** Socio-demographic characteristics of nurses in Jimma zone public hospitals, southwest Ethiopia, 2014

Socio-demographic characteristics		Number	Percent
Working hospital	Jimma University Specialized Hospital	271	85.8
	Shenen Gibe Hospital	20	6.3
	Limu Genet Hospital	25	7.9
Sex	Male	156	49.4
	Female	160	50.6
Marital status	Married	137	43.4
	Single	171	54.1
	Divorced	7	2.2
	Widowed	1	0.3
Working unit/ward	Medical ward	53	16.8
	Surgical ward	66	20.9
	ICU	10	3.2
	Major OR	20	6.3
	Pediatrics ward	42	13.3
	Gyn/obs (maternity)	22	7.0
	Ophthalmology unit	12	3.8
	Psychiatry ward	7	2.2
	OPD	64	20.3
	Chronic illness follow up clinic	20	6.3
Educational qualification	Diploma nurse	197	62.3
	BSc nurse	119	37.7
Total		316	100

*Gyn/obs* gynecology/obstetrics, *OPD* outpatient department

### Respondents’ level of job satisfaction

Data-driven classification system was used to classify the job satisfaction level into low (value less than 54), moderate (values between 54 and 63), and high (values above 63 of the job satisfaction percentage score). Almost one third (33.5%) of the study participants had a low level of job satisfaction (Table 2).

**Table 2:** Level of job satisfaction among nurses working in Jimma zone public hospitals, southwest Ethiopia, 2014

Level of job satisfaction	Number	Percent
Low	106	33.5
Moderate	109	34.5
High	101	32.0
Total	316	100

The overall mean satisfaction of the study participants was 67.43 (SD = 13.85), and the highest level of job satisfaction was satisfaction from coworkers with a percentage mean score of 69.8, while satisfaction from professional opportunities had the lowest percentages mean score of 44.9 (Table 3).

**Table 3:** Percentage mean scores for the summated scales and overall satisfaction (*n* = 316), Jimma zone public hospital, southwest Ethiopia, 2014

Name of subscale	Mean	SD	Minimum (%)	Maximum (%)	Mean (%)
Satisfaction from extrinsic reward	6.31	2.21	25	100	52.58
Satisfaction from scheduling	11.61	3.41	25	100	58.04
Satisfaction from family and work balance	4.95	1.47	25	100	61.87
Satisfaction from coworkers	5.58	1.49	25	100	69.78
Satisfaction from interaction	9.87	2.61	25	100	61.69
Satisfaction from professional opportunity	7.20	2.41	25	100	44.99
Satisfaction from praise and recognition	9.74	2.74	25	100	60.90
Satisfaction from control and responsibility	12.17	3.31	25	100	60.94
Total satisfaction percentage score	67.43	13.85	25.00	91.38	58.13

More than half (59.8%) were very dissatisfied with their salary. Regarding compensation for working weekends and holidays, only 4.7% of respondents were very satisfied. Majority (77.2%) of the respondents were very satisfied on the relation with nursing peers (Table 4).

**Table 4:** Percentage distribution of each item in the job satisfaction scale, Jimma zone public hospitals, southwest Ethiopia, 2014

Items	Very dissatisfied		Moderately dissatisfied		Moderately Satisfied		Very satisfied	
	*n*	(%)	*n*	(%)	*n*	(%)	*n*	(%)
Pay (salary) you receive	189	59.8	69	21.8	46	14.6	12	3.8
Annual leave you receive from the hospital	69	21.8	104	32.9	116	36.7	27	8.5
Sick leave you receive from the hospital	77	24.4	83	26.3	119	37.7	37	11.7
Hours that you work in the hospital	73	23.1	63	19.9	129	40.8	51	16.1
Flexibility in scheduling your working hours	65	20.6	82	25.9	134	42.4	35	11.1
Your satisfaction in your shift rotation	51	16.1	72	22.8	143	45.3	50	15.8
Flexibility in scheduling your weekends off	117	37.0	84	26.6	88	27.8	27	8.5
Compensation for working weekends and holidays	126	39.9	85	26.9	90	28.5	15	4.7
Opportunity for part-time work	116	36.7	92	29.1	79	25.0	29	9.2
Maternity leave time given by the hospital	34	10.8	52	16.5	147	46.5	83	26.3
Interaction with your nursing peers	21	6.6	51	16.1	152	48.1	92	29.1
Interaction with the physicians you work with	47	14.9	86	27.2	134	42.4	49	15.5
Availability of medical equipment/supplies to deliver nursing care in your unit	113	35.8	100	31.6	81	25.6	22	7.0
Satisfaction with the nursing care given to your clients	28	8.9	60	19.0	148	46.8	80	25.3
Opportunities for social contact with your colleagues after work	49	15.5	80	25.3	151	47.8	36	11.4
Opportunities for interact professionally with other disciplines	60	19.0	101	32.0	125	39.6	30	9.5
Opportunities for further education/degree or postgraduate study in nursing	134	42.4	75	23.7	73	23.1	34	10.8
Opportunities to participate in morning rounds	73	23.1	86	27.2	112	35.4	45	14.2
Opportunities to participate in nursing research	210	66.5	70	22.2	28	8.9	8	2.5
Opportunities to write and publish	255	80.7	36	11.4	18	5.7	7	2.2
Recognition from your head nurse	46	14.6	84	26.6	135	42.7	51	16.1
Recognition for your work from superiors	95	30.1	98	31.0	98	31.0	25	7.9
Recognition for your work from peers	35	11.1	64	20.3	152	48.1	65	20.6
Encouragement and positive feedback from matron	100	31.6	93	29.4	86	27.2	37	11.7
Opportunity to make autonomous nursing care decisions	62	19.6	85	26.9	128	40.5	41	13.0
Opportunities for on-the-job training	184	58.2	62	19.6	52	16.5	18	5.7
Responsibility in the unit/ward	40	12.7	69	21.8	134	42.4	72	23.1
Your control over conditions in your working unit	39	12.3	62	19.6	151	47.8	64	20.3
Consideration given to your opinion and suggestions for change in the work setting or office practice	58	18.4	88	27.8	128	40.5	42	13.3

### Respondents’ reasons of satisfaction and dissatisfaction

Over two thirds (68.4%) of the respondents said their main reason for satisfaction in their work was satisfaction in helping clients. Surprisingly, only 8.9% of them reported professional pleasure as their source of satisfaction, whereas 28.9% of nurses mentioned workload as a major source of dissatisfaction; 19.6% stated the performance evaluation process as their major source of dissatisfaction.

### Descriptive result for nurse-physician communication, job-related stress, and professional commitment

Nurse-physician communication-related factors have three subscales which include personal factor with mean score of 8.94, organizational factor with mean score of 21.43, and mutual understanding at work with mean score of 8.91. For job-related stress factors, four subscales were included: workload with a mean score of 8.73, lack of support with a mean score of 6.73, conflict with a mean score of 8.25, organizational decision with a mean score of 6.72, and, lastly, professional commitment with a mean score of 6.49 (Table 5).

**Table 5:** Descriptive result for nurse-physician communication-related factors, job-related stress factors, and professional commitment in Jimma zone public hospitals, southwest Ethiopia, 2014

	Minimum	Maximum	Mean	Std. deviation
Personal factor	3.00	15.00	8.94	3.47
Organizational factor	6.00	30.00	21.43	5.70
Mutual understanding at work	3.00	15.00	8.91	2.87
Workload total score	4.00	16.00	8.73	2.34
Lack of support	3.00	12.00	6.73	2.24
Conflict total	4.00	16.00	8.25	2.69
Organizational decision	3.00	12.00	6.72	1.94
Professional commitment	4.00	16.00	9.46	2.59

### Factors associated with job satisfaction

Candidate predictor variables from bivariate regression were entered into multiple linear regressions. Even though 13 predictor variables were significant (*p* < 0.05) in the bivariate model, only working unit, mutual understanding at work, professional commitment, and workload were found to be the final predictor of job satisfaction. The overall model was significant (*F* = 32.42, *p* < 0.001) and the value of the adjusted *R* ^2^ was 0.37.

When the variance explained by all other variables in the model is controlled, working unit (*β* = 3.08, *p* = 0.032), mutual understanding at work (*β* = 0.78, *p* < 0.001), and professional commitment (*β* = 1.62, *p* < 0.001) showed significant and positive relationship with overall job satisfaction. However, workload showed a significant but negative relationship with the overall job satisfaction score (*β* = −2.66, *p* < 0.001). The rest of the variables were not significantly associated with job satisfaction (Table 6).

**Table 6:** Multivariable linear regression predicting job satisfaction among nurses working in Jimma zone public hospitals, southwest Ethiopia, 2014

Coefficients^a^						
	Unstandardized coefficients		*t*	*p*	95% CI for *β*	
	*β*	Std. error			Lower bound	Upper bound
(Constant)	62.171	4.70	13.23	0.000	52.92	71.42
Working unit						
Outpatient	3.08	1.45	2.13	0.032	0.23	5.93
Inpatient						
Mutual understanding at work	0.78	0.22	3.53	0.000	0.35	1.22
Professional commitment	1.62	0.25	6.61	0.000	1.14	2.10
Workload	−2.66	0.28	−9.54	0.000	−3.21	−2.11
Age (in year)	0.17	0.09	1.86	0.064	−0.01	0.36
Sex	0.29	1.25	0.238	0.812	−2.17	2.77

Max VIF = 1.07, adjusted *R* ^2^ = 0.37

*CI* confidence interval

^a^Dependent variable: total satisfaction score

The model explains 37% of the variance in the outcome variable. A unit increase in professional commitment score would result in the overall job satisfaction score increasing by 1.62 units. A unit increase in the mutual understanding score would result in the overall job satisfaction score increasing by 0.78 units. A unit increase in the workload score would result in the overall job satisfaction score dropping by −2.66 units. And those nurses who were working in the outpatient department (OPD) were 3.08 times more likely to be satisfied than those working in the inpatient unit.

## Discussion

Health professionals especially nurses seem to be less satisfied due to many unidentified factors, and hence, there is a constant threat of attrition. Nowadays in Ethiopia, a number of nurses are leaving their profession to join other non-nursing fields. Therefore, this study set out to assess the level of nurses’ job satisfaction and factors associated with it in Jimma zone public hospitals.

The overall job satisfaction mean score of the respondents was found to be 67.43 (SD = 13.85). And almost one third (33.5%) of the study participants had a low level of job satisfaction. This finding is comparable with a study done in Islamabad [23] which showed that 37.14% of the participants had a low level of job satisfaction. Methodology difference among the studies might have contributed to the result. However, it is lower than the findings of studies conducted in Australia [24], California [25], and India [26] where nurses were highly satisfied. This might be due to difference in socio-economic status and organizational policies including staff handling and infrastructure between the study setups.

Nurses were most satisfied with their coworkers in different studies of Italy [3], Lebanon [27], Jordan [28], and the USA [29] which is consistent with the current study, as the highest level of satisfaction reported was satisfaction from coworkers.

Respondents’ least satisfaction was from professional opportunities and extrinsic reward which is in line with studies done in Italy [3] and Kuwait [6] but inconsistent with reports from the USA [29], Lebanon [27], and Jordan [28] that showed nurses with higher satisfaction with regard to career development. This discrepancy might be due to differences in institutional policies of the study setups that lead to substantial difference in opportunities for further education. It is clear that career development, long-term learning, and training activities in nursing promote job satisfaction, increase retention of nurses, and enable continued provision of quality care [30].

Praise and recognition is expected to motivate nurses and make them happy at work [21], but in this study, nurses stated administration from nursing management as a source of dissatisfaction. This is inconsistent with a study done in India [31] where most of the supervisors and matrons were reported to be very cooperative. Majority (59.8%) of the respondents were also least satisfied with their salary which is consistent with studies from Turkey [32] and Australia [23] in which around 60% of nurses were least satisfied with their pay.

Nurses in hospitals today provide care for an increasing number of patients with chronic conditions [33], and these cases are managed at the inpatient unit. This can increase the work burden of nurses working in the inpatient unit and make them less satisfied. The finding of the present study also revealed that nurses working at the inpatient unit were less satisfied. This is consistent with a study done in Jordan [28] where nurses working in inpatient units deal with more severely ill patients and were less satisfied. Workload also predicted job satisfaction negatively. Many respondents also mentioned workload as a reason to be less satisfied, which is consistent with studies done in the USA [34] and Taiwan [35] that reported workloads’ significant negative relation to job satisfaction.

Professional commitment was found to have an effect on nurses’ job satisfaction; which is in line with studies done in China [36–38] that revealed professional commitment as a strong predictor for job satisfaction. Individuals with high levels of professional commitment are more likely to engage in activities that favor their organization [39].

In this study, mutual understanding predicts a nurse’s job satisfaction positively. This is consistent with studies done in Iran [40] and the West Coast region [41] where nurse-physician relationships had impact on nurses’ satisfaction. When there is mutual understanding at work, there is a sense of recognition and feeling of respect between them, which further brings satisfaction at work.

Diploma nurses were more satisfied than degree nurses. This finding is in line with studies done in the USA [42] and Kuwait [6] that proved nurses with higher levels of education were less satisfied with their occupation. A probable reason could be that more educated employees have higher levels of expectations which health care institutions are often unable to fulfill.

More than two thirds (69%) of nurses stated helping clients as a source of satisfaction at work; this result is in line with a studies done in South Africa (76%) [43] and Malawi (73%) [44], where participants reported helping the sick as a source of satisfaction.

### Limitations

The information gained from participants is based upon their subjective perceptions, and it is possible that they either over- or underreported their level of satisfaction. By assuming that nurses can read and understand the English language, there was no need of translating the questionnaire into the local language and this may possibly cause some misunderstanding in some words or terminology.

## Conclusions

One third of the study participants had a low level of job satisfaction. Level of job satisfaction was positively associated with mutual understanding at work and professional commitment, whereas it was found to be negatively associated with working at the inpatient unit and increased workload.

Fostering and maintaining a higher level of job satisfaction among nurses is vital to limit turnover and ensure the delivery of quality care.

## Abbreviations

JUSH: Jimma University Specialized Hospital
MDGs: Millennium Development Goals
MMSS: McCloskey/Mueller Satisfaction Scale
OPD: Outpatient department
